# Anti Diabetic effect of CL 316,243 (A β3-Adrenergic Agonist) by Down Regulation of Tumour Necrosis Factor (TNF-α) Expression

**DOI:** 10.1371/journal.pone.0045874

**Published:** 2012-10-04

**Authors:** Masoud Ghorbani, Mehdi Shafiee Ardestani, Sedigheh Hatami Gigloo, Reza Ahangari Cohan, Davoud Nouri Inanlou, Peyman Ghorbani

**Affiliations:** 1 Department of Microbiology and Immunology, Pasteur Institute of Iran, Tehran, Iran; 2 Research and Production Complex, Pasteur Institute of Iran, Karaj, Iran; 3 Department of Biochemistry, Microbiology and Immunology, University of Ottawa, Ottawa, Ontario, Canada; 4 Department of Hepatitis and AIDS, Pasteur Institute of Iran, Tehran, Iran; 5 Department of Laboratory Medicine and Pathobiology, University of Toronto, Toronto, Ontario, Canada; The Chinese University of Hong Kong, Hong Kong

## Abstract

**Objective:**

Obesity is a risk factor for the development of insulin resistance and is one of the most important contributors to the pathogenesis of type2 diabetes, which acts mainly through the secretion of adipokines such as TNF-*α* that may influence insulin sensitivity. TNF-*α* affects many aspects of adipocyte function, such as adipocyte development and lipid metabolism.

**Material and Methods:**

We demonstrated that there is a correlation between the expressions of TNF-α in retroperitoneal WAT and insulin-resistance in 8 genetically obese *fa/fa* rats. Treatment of animals with CL 316,243, a β3-adrenergic agonist, showed an improvement of insulin-resistance that was linked with the suppression of TNF-α mRNA expression in WAT.

**Results:**

These results confirm the association between TNF-α expression and the insulin-resistant condition in rats. Our finding indicates that the hyperglycaemia and hyperinsulinemia induced by insulin-resistance correlated positively with the expression of TNF-α mRNA in an abdominal WAT depot.

**Conclusion:**

We conclude that CL 316,243 possesses both anti-diabetic effects and anti-obesity effects in rodents.

## Introduction

Insulin resistance is an important pathogenesis in the development of obesity. It is related with glucose dysfunction and other disorders, including but not limited to endothelium dysfunction, fatty liver and atherosclerosis [1]. TNF-α is a cytokine expressed by both macrophages and adipocytes and is known to induce insulin resistance associated with hyperinsulinemia by impairment of insulin action on peripheral glucose uptake and hepatic glucose output [2], [3]. This impairment of insulin action is suggested to occur because of the phosphorylation of insulin receptors and IRS-1 by TNF-α [4].

In the previous studies we demonstrated that treatment with CL 316,243, a highly selective agonist for β3-Adrenergic Receptor partially reversed obesity in *fa/fa* Zucker rats [5]. Other studies have previously shown that CL-treatment improves insulin-resistance in KKA mice [6]. Since the role of TNF-α in inducing insulin-resistance in obese animals is now well-known [7] we hypothesised that CL-induced improvement in insulin-resistance might be associated with the suppression of TNF-α mRNA expression in adipose tissue. To test this hypothesis, two groups of rats (*fa/fa* and lean Zucker rats) treated with either CL 316,243 or saline were used to demonstrate the effect of CL 316,243 on the expression of TNF-α mRNA as well as blood glucose and serum insulin levels.

## Materials and Methods

All animal protocols were reviewed and approved by the University of Ottawa Animal Care Protocol Review Committee; protocol number is CHEO-60.

Twenty-four lean and 24 genetically obese *fa/fa* rats were purchased at 11–12 weeks of age and housed at 24° in plastic cages with wood chip bedding and with free access to food (Agway R-M-H 4020 chow, 14.5% energy from fat) and water until they reach 30 weeks of age. At this time one group of eight of each genotype was killed (week 0). Two groups of eight from each genotype were then implanted with osmotic minipumps (Alzet 2002, Alza, Palo Alto CA) under Isoflurane anesthasia and received 0.49 µl per hour containing either saline or CL316,243 (daily dose was 1 mg/kg body weight) for the next 4 weeks. Pumps were replaced after two weeks. Body weights and food intakes were measured weekly. Rats were killed by decapitation. Retroperitoneal white adipose tissue (RWAT) and epididimal WAT depots were removed and weighed. Although both retroperitoneal and epididymal WAT depots were studied, only data for retroperitoneal WAT (RWAT) are presented, because changes in epididymal WAT were similar although of lesser magnitude. Blood samples were collected either by cannulating the tail vein at the beginning of the study or at the time of decapitation at the end of the study.

In a parallel study, another 16 lean and 16 genetically obese *fa/fa* rats (8 rats per group) were placed in plastic cages with free access to food and water until reach the age of 30 weeks. They were also implanted with osmotic minipumps releasing either saline or CL 316,243 for 4 weeks as described before. All rats were then given 2 mg glucose/g body weight by gavage for oral glucose tolerance test after overnight fasting and blood samples were taken from the tail vein before and at 60 and 120 min after glucose administration. Blood samples were then tested for blood glucose, serum insulin and fatty Acids (FAs) concentrations as described here.

### Measurement of Blood Glucose, Serum Insulin FAs

Serum insulin was measured by ELISA (Ultra Sensitive Rat Insulin kit, Morinaga Institute of Biological Science, Inc). Serum glucose was measured by a hexokinase/glucose-6-phosphate dehydrogenase method on a Coulter DACOS device. Homeostasis model assessment was calculated as the product of fasting insulin (microunits per milliliter) and fasting glucose (micromoles per liter) divided by 22.5 [8]. Fasting serum insulin and homeostasis model assessment were used as markers of insulin resistance [9]. Serum FAs was measured using a FAs assay kit (Roche, Indianapolis, IN).

### RNA Isolation and Northernblot analysis

Total RNA from homogenized adipose tissue was extracted using a Qiagen RNeasy Lipid Tissue Mini Kit (Cat # 74804) according to the manufacturer's instruction (Qiagen, USA) and subjected to Northern Blot analysis as described in standard protocols. Briefly, twenty micrograms of RNA from each tissue was electrophoretically fractionated through 1.2% agarose gels containing 3% formaldehyde buffered in 20 mM 3-(N-morpholino) propanesulfonic acid and 1 mM ethylenediaminetetraacetic acid at pH 7.4. The RNA was transferred to Zeta-Probe GT nylon membranes (Bio-Rad Laboratories, Hercules, CA) overnight and fixed by ultraviolet cross-linking. A ^32^P-labelled TNF-α probe was prepared from rat complementary DNA using DECA prime II Random-Primed DNA Labeling Kit and NucAway Spin Columns (Ambion, Austin, TX). Hybridization and washing were performed at 65°C as described by Church and Gilbert (Church and Gilbert 1984). Membranes were exposed to Blue Lite Autorad Film (ISC Bio Express, Kaysville, UT) in the presence of an intensifying screen at −80°C. Kodak 1D Image Analysis Software, version 3.6 (Carestream Health, New Haven, CT) was used to measure intensity of the bands for statistical analysis.

### Statistical Analysis

Results are presented as means ± SEM. Statistical analysis used Instat software to do ANOVA followed by a Student Newman-Keuls post hoc test. Significance differences are based on *P*<0.05.

## Results

**Figure 1:**
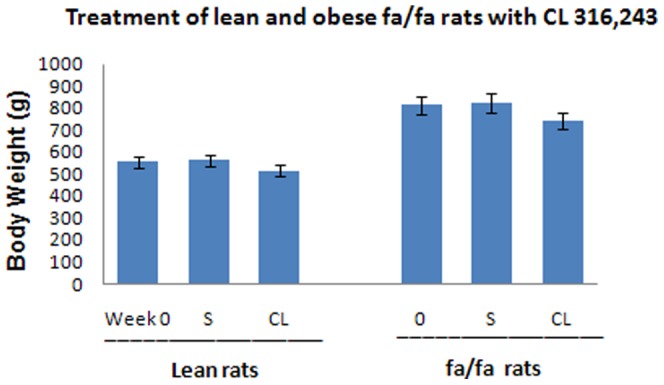
Body weight of both Lean and *fa/fa* rats. Body weight was measured at week 0 and after 4 weeks treatment with either saline (S) or CL316,243 (CL). *Significant effect of CL compared with pretreated state at week 0.

**Figure 2:**
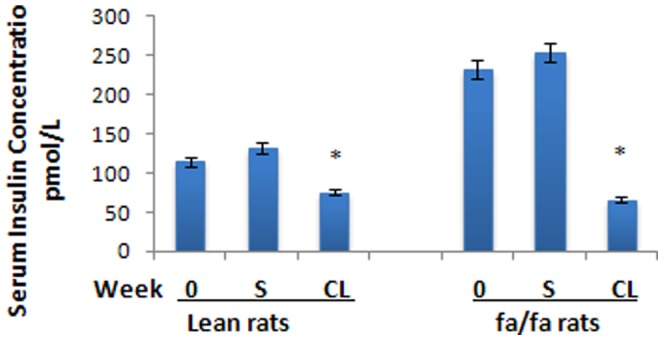
Serum insulin concentration. CL-*fa/fa vs* S-*fa/fa* after 4 weeks of treatment, *P<0.001*. CL-lean *vs* S-lean after 4 weeks of treatment, *P<0.001*. Serum insulin level is higher in *fa/fa* rats than in lean rats at week 0 and in S-*fa/fa*, *P<0.001*. Serum insulin level decreased significantly in CL-lean as compared with S-lean, *P<0.001*. There is no significant difference between CL-*fa/fa* CL-lean rats after 4 weeks treatment.

**Figure 3:**
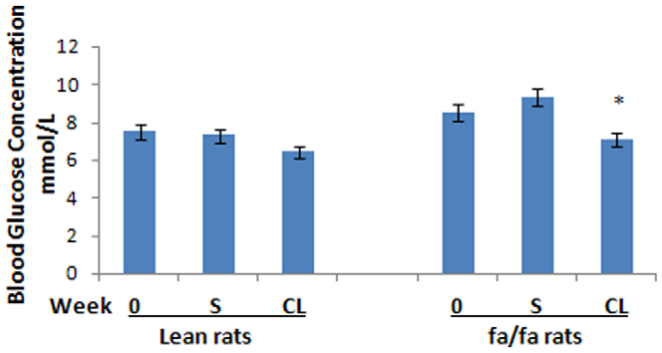
Serum glucose concentration. CL-*fa/fa vs* S-*fa/fa* after 4 weeks of treatment, *P<0.001*, and *vs fa/fa* week 0, *P<0.05*. CL-lean *vs* lean at week 0 and S-lean after 4 weeks of treatment, is not significant. Glucose is higher in *fa/fa* than lean at week 0, *P<0.05*, and in S-*fa/fa* than in S-lean after 4 weeks of treatment, *P<0.001*. There is no significant difference between CL-*fa/fa* and CL-lean after 4 weeks of treatment.

In this experiment both retroperitoneal and epididymal WAT depots were studied. Since changes in epididymal WAT were similar to retroperitoneal WAT (RWAT), data are reported only for RWAT. Wet weight of RWAT was almost 10 times higher in fa/fa rats than in lean rats and was reduced by 35% by the CL-treatment (Table 1). The weight of RWAT was also reduced by the CL-treatment in the lean rats (week 0 *vs* week 4) (Table 1).The body weight of *fa/fa* rats was highly variable, much more than that of the lean rats. Since the variation of body weight in *fa/fa* rats was very high, the decrease in mean body weight of these rats induced by the CL-treatment did not reach statistical significance (Figure 1; Table 1). However, the *fa/fa* rats lost an average of 62±19.8 grams in four weeks of treatment. CL-treatment reduced abdominal fat content in both *fa/fa* and lean rats (Table 1).The average fat content of RWAT depots was higher in *fa/fa* rats than in lean control animals (Table 1). CL 316,243-treatment decreased the fat content of these abdominal depots in both lean and *fa/fa* rats. Before treatment, serum insulin levels were significantly higher in obese and insulin-resistant *fa/fa* rats than in lean rats (Figure 2). Treatment with CL significantly decreased the serum insulin level in these animals (Figure 2). Blood glucose level was also slightly higher in obese animals than lean rats (Figure 3). After treatment with CL, the level of blood glucose was significantly decreased in *fa/fa* rats (Figure 3).

**Table 1:** Body weight and retroperitoneal WAT (RWAT) measurement in rats.

Groups	Body weight	Weights of RWAT
Lean rats (week 0)	553±20.88	6.04±0.6
Saline-lean (week 4)	561±18.5	5.21±0.65
CL-lean (week 4)	513±14.4	2.23±0.23*
*fa/fa* rats (week 0)	812±45	48.9±6.57
Saline-*fa/fa* (week 4)	823±28	51.84±6.38
CL-*fa/fa* (week 4)	743±35	31.9±4.13*

Values are mean ±SEM for the number of rats (8). Treatment with saline or CL was between week 0 and week 4.

* Signifcant effect of CL compared with saline-treated rats of the genotype.

**Figure 4:**
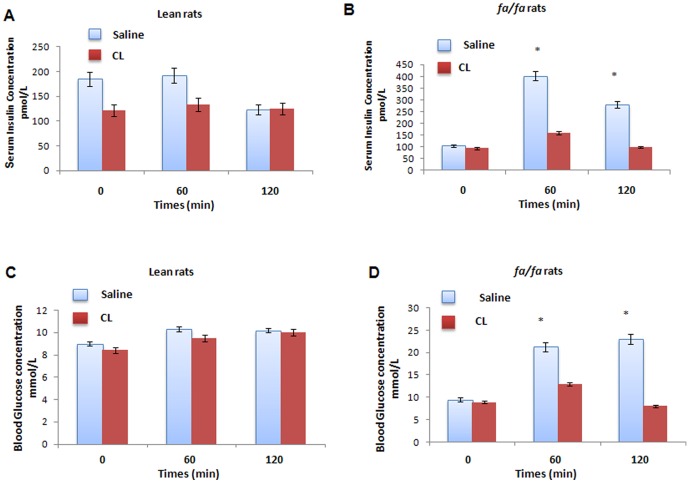
Effect of CL 316,243 on serum insulin (A and B) and blood glucose (C and D) responses during the oral glucose test in the lean and *fa/fa* rats treated with either saline or CL. *P<0.05, compares the significant differences between the two groups of lean and *fa/fa* rats.

In oral glucose tolerance test, serum insulin and blood glucose levels were significantly higher in the fatty rats treated with saline after feeding with 2 mg glucose/g body weight as compared with lean rats treated with saline (Figure 4A and C). Treatment with CL, on the other hand, decreased the both serum insulin and blood glucose levels in only fatty rats significantly (Figure 4B and D).

**Figure 5:**
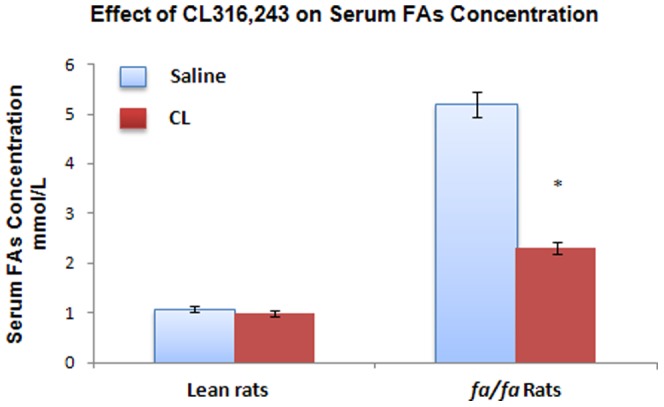
Effect of CL 316,243 on serum FAs concentration. *P<0.05, compares the significant differences between the two groups of lean and *fa/fa* rats.

To determine whether β_3_-adrenergic responsiveness is intact in fa/fa rats, we measured serum FAs after 4 weeks of treatment with CL 316,243 stimulation. Serum FAs in fa/fa rats was approximately 5 times greater than that in lean rats. Four weeks of treatment with CL significantly decreased the serum FAs level, although not entirely to control values (Figure 5).

**Figure 6:**
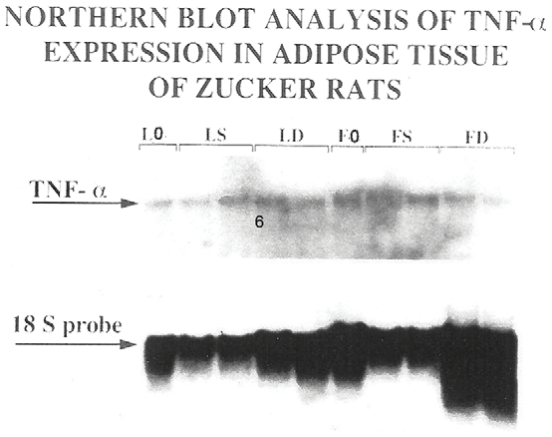
Northern blot analysis of TNF-α mRNA expression in the RWAT of lean and *fa/fa* rats. Northern hybridization of TNF-α mRNA and 18S ribosomal RNA (as control) was performed using the standard protocol. Briefly, total RNA from retroperitoneal WAT was extracted by Chomczynski method (Chomczynski 1993). Total RNA (∼20 µg) was denatured and separated on formaldehyde gel. Lane 1, L0, lean-pretreatment at week 0; lanes 2 and 3, S-lean; lanes 4 and 5, CL-lean; lane 6, F0, *fa/fa*-pretreatment at week 0; lanes 7 and 8, S-*fa/fa* and lanes 9 and 10, CL-*fa/fa* rats.

**Figure 7:**
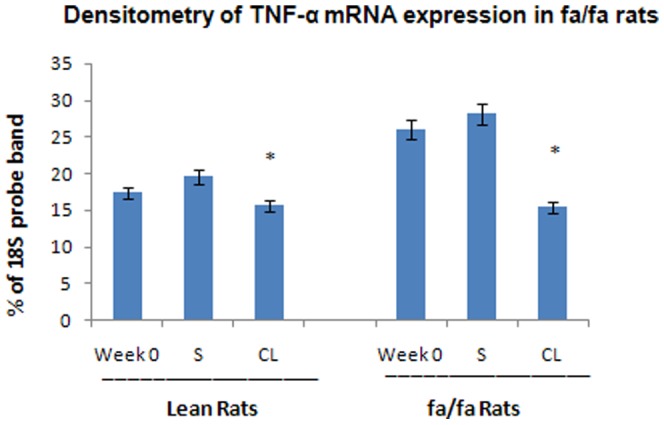
Densitometry of the TNF-α mRNA bands as compared to 18S bands in the same lane. All values are means± SEM. There are significant differences as follow: CL-*fa/fa vs* S-*fa/fa*, *P<0.001*. CL-*fa/fa vs* S-lean, *P<0.05*. CL-lean *vs* S-lean and lean week 0, is not significant. There is nosignificant difference between CL-*fa/fa* and CL-lean rats at 4 weeks after treatment.

These results showed that treatment with CL 316,243 improved insulin-resistance in *fa/fa* rats, allowing normoglycemia at a normal concentration of insulin in the blood. The level of TNF-α mRNA expression in RWAT was measured in both groups of animals. The expression of TNF-α mRNA in *fa/fa* rats was not significantly different with the lean rats. However, treatment with CL 316,243 significantly decreased the expression of TNF-α in WAT of obese Zucker rats (Figures 6 and 7).

## Discussion

Elevated TNF-α mRNA expression in WAT usually correlates with massive obesity and insulin resistance [10]–[12]. Elevated TNF-α mRNA expression has been shown in ob/ob, tub/tub, and KKA mice, and Zucker *fa/fa* rats [13] as well as transgenic model of obesity/insulin resistance created by ablation of brown adipose tissue (BAT) via a bacterial toxin gene driven by the UCP promoter [4], [14]. It is possible that adipose tissue is able to generate mediators that influence the activity of insulin in various target tissues [11]. A significant decrease in serum FAs level is also believed to be correlated with an increase in fatty acid oxidation in the brown adipose tissue and skeletal muscles as reported in chronic CL316243 treatment of MKR mice, in contrast to acute treatment with CL that increases the FAs mainly due to the activation of lypolysis [15].

The main goal of the present studies was to determine whether treatment of insulin resistant fatty Zucker rats with CL 316,243 improves both obesity and insulin resistance. In this experiment we have demonstrated the correlation of expression of TNF-α in RWAT and insulin-resistance in genetically obese *fa/fa* rats, and that the improvement of NIDDM in these animals by treatment with CL 316,243 is linked with the suppression of TNF-α mRNA expression in WAT. These results confirm the association between TNF-α expression and the insulin-resistant condition in rats as shown by other investigators. Taken together, our finding indicated that the hyperglycaemia and hyperinsulinemia induced by insulin-resistance correlated positively with the expression of TNF-α mRNA in an abdominal WAT depot. Others have shown that chronic infusion of CL in lean and obese diabetic rats improves glucose tolerance and insulin resistance in obese-diabetic rats associated with a marked increase in glucose uptake in BAT and WAT [16]. Other studies have demonstrated the effect of CL 316,243 on recovering the reduction of adiponectin and adiponectin receptor mRNA expression in epididimal white adipose tissue in KKAy mice along with the suppression of TNF-α mRNA in both epididimal white adipose tissue and brown adipose tissue of KKAy mice [17]. It was also suggested that the normalization of adiponectin and its receptors together with TNF- α may result in the amelioration of obesity in insulin resistant obese animals [17]. These data and our findings confirm the idea that CL 316,243 may not only possess anti-obesity effects, but also has anti-diabetic effects and consequently could be useful for treating obesity as well as NIDDM in obese animals.
